# Effects of Local Anesthetics on Liposomal Membranes Determined by Their Inhibitory Activity of Lipid Peroxidation

**DOI:** 10.1021/acs.molpharmaceut.2c01053

**Published:** 2023-04-27

**Authors:** Yusuke Horizumi, Satoru Goto, Miwa Takatsuka, Hideshi Yokoyama

**Affiliations:** Faculty of Pharmaceutical Sciences, Tokyo University of Science, 2641 Yamazaki, Noda, Chiba 278-8510, Japan

**Keywords:** “lipid membrane”, “local anesthetics”, “singular value decomposition”, “lipid peroxidation”, “antioxidants”

## Abstract

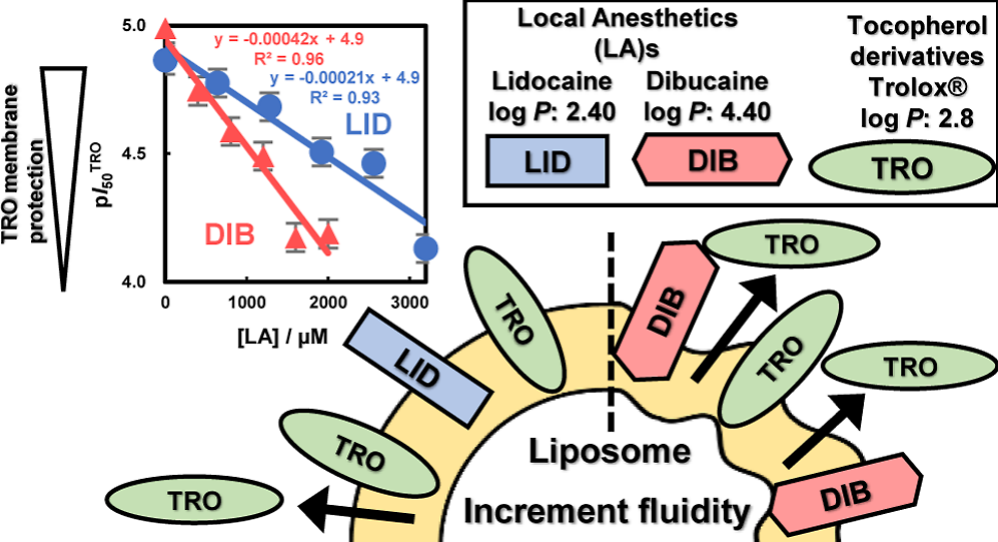

In this study, we investigated the effects of drugs on membrane function in which lipid peroxidation was inhibited by the antioxidant Trolox (TRO) in liposomes containing egg yolk lecithin. Local anesthetics (LAs), such as lidocaine (LID) and dibucaine (DIB), were used as model drugs. The effect of LAs on the inhibitory activity of TRO was evaluated by calculating the p*I*_50_ from the inhibition constant *K* calculated by curve fitting. p*I*_50_^TRO^ indicates the strength of TRO membrane protective function. p*I*_50_^LA^ indicates the strength of LA activity. LAs inhibited lipid peroxidation in a dose-dependent manner and decreased p*I*_50_^TRO^. The effect of DIB on p*I*_50_^TRO^ was 1.9 times more than that of LID. This result indicated that LA may improve the fluidity of the membrane, which may facilitate the migration of TRO from the membrane to the liquid phase. As a result, TRO is less likely to suppress lipid peroxidation within the lipid membrane, possibly resulting in a decrease in p*I*_50_^TRO^. The effect of TRO on p*I*_50_^LA^ was found to be similar in both, indicating that it did not depend on the type of the model drug. These results suggest that our developed procedure successfully quantified the effects of LAs on lipid membrane functions. We were able to obtain the characteristics of model drugs independent of TRO by simultaneously measuring and analyzing the lipid peroxidation inhibitory activities of TRO and model drugs in liposomes.

## Introduction

Maintaining life activities requires oxygen and consumes energy. The sequential reduction of oxygen in the body leads to the formation of highly reactive oxygen species (ROS).^1^ Excessive production of ROS causes oxidative damage to the heart and cerebral blood vessels.^2^ Cell membranes are rich in polyunsaturated fatty acids (PUFAs), which make them susceptible to ROS damage, which is also referred to as “lipid peroxidation”.^3^ The occurrence of lipid peroxidation has been linked to aging and cancer development and is therefore of interest in future pharmacological treatments.^4^

Lipid peroxidation is a complex process that occurs in both plants and animals.^5^ It involves the formation and propagation of lipid radicals, oxygen uptake, and the rearrangement of double bonds in unsaturated lipids. Eventually, lipid peroxidation may lead to lipid membrane destruction, producing various breakdown products, such as alcohols, ketones, aldehydes, and ethers.^5^ Biological membranes are often surrounded with unsaturated fatty acid-rich, oxygen-rich, and metal-rich fluids.^5^ Lipid peroxidation begins with the withdrawal of hydrogen atoms from unsaturated fatty acids to form lipid radicals.^5^ When lipid endoperoxides containing at least three methylene-interrupted double bonds are present in unsaturated fatty acids, malondialdehyde (MDA) may be formed as a breakdown product.

This lipid peroxidation reaction can be chain-stopped by various free-radical scavengers.^6^ A typical example of a radical scavenger in the body that inhibits lipid peroxidation is α-Tocopherol (α-Toc).^7^ α-Toc inserted into the cell membrane reacts with ROS and the peroxy lipid radicals generated in the body to form stable α-Toc radicals.^8^ This reaction inhibits the lipid peroxidation cascade caused by oxidative stress. Many anti-inflammatory drugs and antioxidants have been reported to inhibit lipid peroxidation.^9^ Previous studies have found that the degree of lipid peroxidation is correlated with the fluidity of lipid membranes.^10^ However, limited research has been conducted to determine the extent to which drugs that affect lipid membrane fluidity are involved in inhibiting lipid peroxidation.

An example of a drug that increases the fluidity of lipid membranes is local anesthetic (LA). It is specifically used in modern surgical practices to reduce pain. Currently, the molecular mechanism of LA has been partially described as a direct interaction between the anesthetic and an ion channel protein that allows ions to move across the cell membrane.^11^ Because most LA molecules have large hydrophobic moieties, they can interact with hydrophobic regions in the cell membrane, altering their physical properties.^12,13^ As a result, channel proteins in cell membranes are considered to be indirectly affected by LAs due to changes in membrane physical properties.^14^ Therefore, LAs have been extensively studied as drugs that alter the fluidity of lipid membranes. Lidocaine (LID), a type of LA, has been reported to solubilize lipid membranes at high concentrations, resulting in neurotoxicity.^15^ Dibucaine (DIB) is another type of LA that performs similar functions as LID. Therefore, LID and DIB are suitable as model drugs to alter the fluidity of lipid membranes.

However, cell membrane complexity makes it difficult to investigate lipid raft stability after the addition of LAs. Thus, liposomes regarded as simple cell membrane models are widely used to reveal the interactions between lipid rafts and additive molecules, such as LAs. In this study, egg yolk lecithin (EyPC) liposomes were used to mimic the plasma cell membrane. Notably, PUFAs are abundant in EyPC.^16^ EyPC has been widely used in the study of lipid peroxidation using model membranes.^17−21^

The focus of this study was to investigate the effects of LA, a drug that increases the fluidity of lipid membranes, on the inhibition of lipid peroxidation occurring in the EyPC bilayer using the 2-thiobarbituric acid reactive species (TBARS) method. The spectra, peaks, absorption, and signals generated by the TBARS method, which were due to dyes not produced from lipid peroxidation, were separated by a singular value resolution. This could explain the effects of LAs on cell membrane functions and the characteristics of each LA drug.

## Materials and Methods

### Materials

EyPC, LID, DIB, Trolox (TRO), 1,1-diphenyl-2-picrylhydrazyl free radical (DPPH), hydrogen peroxide (H_2_O_2_), sodium dodecyl sulfate, 2-thiobarbituric acid (TBA), and 2,6-di-*tert*-butyl-p-cresol (BHT) were purchased from the Tokyo Chemical Industry. 1,1,3,3-Tetraethoxypropane, chloroform, and diethyl ether were purchased from FUJI-FILM Wako Pure Chemical Corporation (Osaka, Japan). Ethanol (95%) was purchased from Kanto Chemical Co., Inc.(Tokyo, Japan). All other reagents used were of the highest commercially available grade.

### Preparation of Liposomes

Multilamellar liposomes (MLVs) were prepared as described previously.^22^ EyPC in diethyl ether solution was evaporated on a lipid film for 90 min and dried in a desiccator for 18 h. The dried lipid film was placed in a solution composed of 0.14 M NaCl, 8.9 mM Na_2_HPO_4_, 1.5 mM KH_2_PO_4_, and pH7.6 (D.PBS) by continuous permeation with a shaker at 300 rpm at 50 °C for 30 min. MLV suspension was sonicated in a sonicator at 50% amplitude with a 50% duty cycle for 60 min. This sonication treatment produced liposome suspensions containing mainly small unilamellar liposomes (SUVs). Total phosphorus concentration was measured as described previously.^23^ The average particle sizes (standard deviation) for MLVs and SUVs were 256.7 nm (143.3) and 170.7 nm (64.2), respectively.

### Lipid Peroxidation

Lipid peroxidation in liposomes (phosphorus concentration 1.3 μg/μL) was induced by the addition of 0.2 mM Fe(NH_4_)_2_(SO_4_)_2_ and 0.1 mM H_2_O_2_ at 37 °C in D.PBS/ethanol = 9:1 (molar ratio) for 12 min and was stopped by further addition of 2% w/v BHT.^24^ Hereafter, 0.2 mM Fe(NH_4_)_2_(SO_4_)_2_ and 0.1 mM H_2_O_2_ are denoted to as Fenton’s reagent (FR).

### Spectrophotometry

The degree of peroxidation, represented by the number of TBA-reactive substances produced, was estimated by the TBARS method.^5^ A standard curve was constructed using tetraethoxypropane, a precursor of MDA. The TBARS spectrum was measured by a V-750 UV–vis spectrophotometer (JASCO, Japan). All measurements were performed at 25 °C.

### ESR Measurement

Electron spin resonance (ESR) measurements were performed according to the method proposed by Takatsuka et al.^25^ All experiments were performed at 25 °C. The ESR spectra were recorded by a micro ESR (Bruker, Germany) 30 min after mixing the sample with each radical species, which was the endpoint of the reaction. The DPPH radical-scavenging ability of each selected TRO was determined using ESR spectroscopy. An aliquot of 100 μL of TRO solution in 95% ethanol/water = 4:1 (v/v) at different concentrations was mixed with 200 μL of DPPH in 95% ethanol/water = 4:1 (v/v) and 200 μL of LID or DIB solution in 95% ethanol/water = 4:1 (v/v). All reaction mixtures contained 0.5 mM DPPH, a TRO solution at each concentration, and 16 mM LID or 10 mM DIB. The control solution did not contain TRO or LA.

### SVD Procedure

Singular value decomposition (SVD) is a method that is deeply rooted in linear algebra.^26^ The *i*th observed UV–vis spectrum and ESR spectrum {|1 ≤ *i* ≤ *n*} are represented as *n* m-dimensional vertical vector that is measured at the wavelength {|1 ≤ *j* ≤ *m*}. Matrix *M* comprises a horizontal sequence of vectors from the first spectral vector through the *i*th spectral vectors, with an *m* × *n* rectangular matrix that is defined as follows, where *m* ≥ *n,*
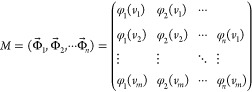
1In the above equation, *M* and *M*^*t*^ are the real and transposed matrices, respectively. Their products *M*^*t*^*M* and *MM*^*t*^ become orthogonal matrices, and the eigenvectors are the rows of *U* and *V*, respectively. The matrices describing *M* are transformed into the following formula,
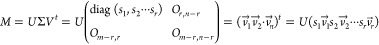
2The diagonal matrix *E* represents the diagonal elements {|1 ≤ *i* ≤ *r*} of the positive real values in descending order. These elements are singular values, indicating dispersion.^27^ The *i*th column of the orthogonal matrix *V* is the coefficient vector corresponding to the singular value *s*_*i*_, and vector  is known as a singular vector. The principal component vector  is the coefficient vector  multiplied by the corresponding singular value *s*_*i*_
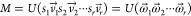
3The matrix *U* consists of rows that are the basis function vectors.^28^ We practically determined the dimensionality according to the diagram for the logarithm of the singular values in descending order versus the indices corresponding to the documental spectra, that is, the minimum dimensionality of the basic functions required to reproduce the vector space of the documental spectra. This could be practically negligible with a singular value less than several hundredths of the highest singular value of the first principal components. Because the dimensionality *r* is determined under this criterion instead of the mathematical rank ρ, the yielded principal components approximately reproduced the vector space, including the documental spectrum as the *j*th feature vector  composed of the *i*th elements *x*_*i*,*j*_,
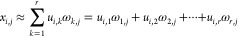
4

### Calculation of p*I*_50_

Curve fitting was performed using the following equation with ω calculated from the SVD, which is the sum of the first and second principal components:
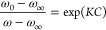
5where ω_0_ is the sum of ω values at the initial concentration. ω_∞_ is the sum of ω values at the final concentration. ω is the sum of ω values at each concentration, *K* is the inhibition constant, and *C* indicates the concentration of each drug used. The effect of LAs on the inhibitory activity of TRO was evaluated by calculating the p*I*_50_ from *K* calculated by curve fitting. p*I*_50_ was calculated using the following equation.
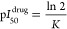
6

## Results and Discussion

### UV–Vis Spectra of Lipid Peroxidation Occurring by FRs

**Figure 1:**
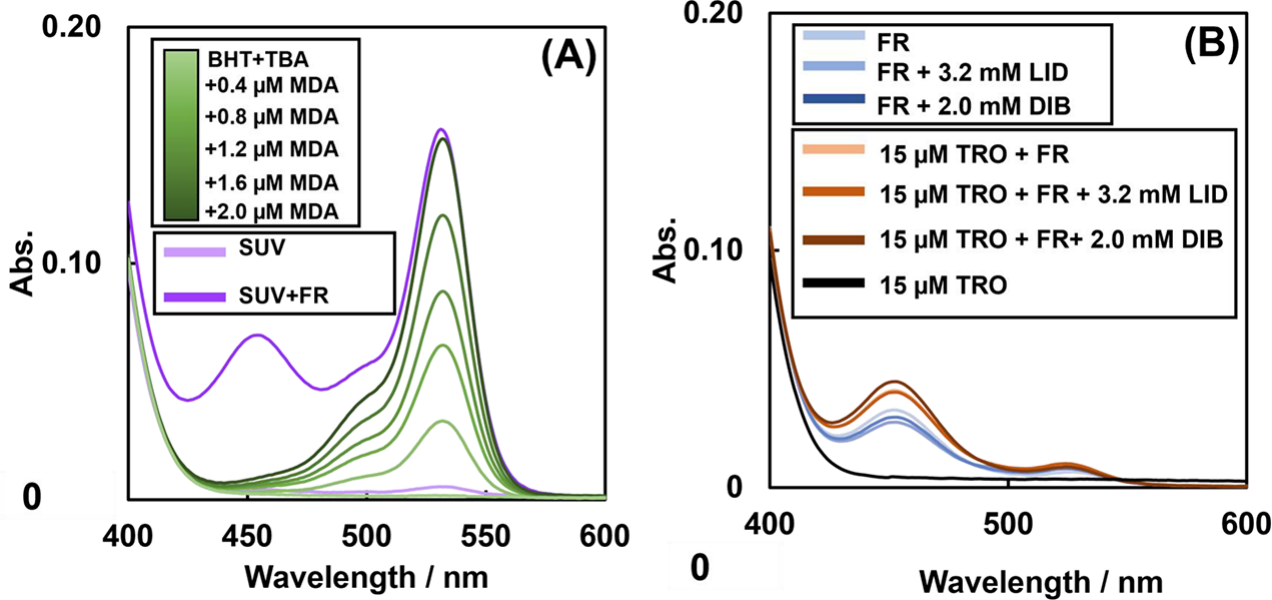
(A) TBARS spectra of MDA solutions at various concentrations and SUV suspensions in which lipid peroxidation occurred. (B) TBARS spectra of solutions containing each drug. These solutions do not contain SUVs. The solutions are 10% ethanol in D.PBS. SUV means 1.3 μg/mL phosphorus concentration liposome suspension. FR means that [Fe(NH_4_)_2_(SO_4_)_2_] = 0.2 mM and [H_2_O_2_] = 0.1 mM are added to the solution.

FRs are combinations of hydrogen peroxide and divalent iron ions. Hydrogen peroxide catalyzes the FR with divalent iron ions to produce hydroxyl radicals. This reaction is responsible for the generation of majority of the hydroxyl radicals in the body. To confirm whether FR can induce lipid peroxidation, we measured the spectra of the supernatant of TBARS generated by the reaction of SUV + FR with TBA. It is often claimed that the TBARS method is nonspecific.^29^ There are also reports that nonlipid peroxide substances react with TBA.^30^ However, in separation analysis, including high-performance liquid chromatography (HPLC), multiple dyes with absorbance at 530 nm are separated, and fine peaks are lost at the detection limit. Therefore, quantification is not guaranteed. In this study, since the purpose is to quantify the concentration dependence of the reagent, we performed UV–vis spectrum measurement that can quantify the total amount. On this background, our previous studies have reported the use of UV–vis spectroscopy rather than HPLC to trace reactions, including intermediates, in the spectroscopic analysis of compounds.^31^ The spectra of the supernatant reacted with various concentrations of MDA and TBA as standards for TBARS were also measured. The spectra increased only when SUV + FR and MDA were included. Figure 1A shows that the absorbance at 530 nm increased specifically in both MDA and SUV + FR samples Therefore, the absorbance at 530 nm indicates the degree of lipid peroxidation. Therefore, the absorbance at 530 nm indicates the degree of lipid peroxidation. However, the absorbance at 455 nm was also increased in the presence of SUV + FR. The spectra of the supernatant, which included the reaction of FR and TBA, were measured. Figure 1B shows that the absorbance at 455 nm increased for both the FR-only and TRO + FR samples. Furthermore, the absorbance at 530 nm was also increased by the TBARS method, indicating that the products of SUV + FR, FR-only, and TRO + FR react with TBA to simultaneously generate a dye that is not derived from lipid peroxidation. Therefore, peaks at 530 and 455 nm are generated by lipid peroxidation. In addition, in Figure 1B, the formation of dyes at 455 nm unrelated to lipid peroxidation was also confirmed. These results suggest that the dye at 455 nm contains not only the peaks of lipid peroxidation but also dye peaks not derived from lipid peroxidation.

### Effects of LA and TRO on Lipid Peroxidation, Respectively

**Figure 2:**
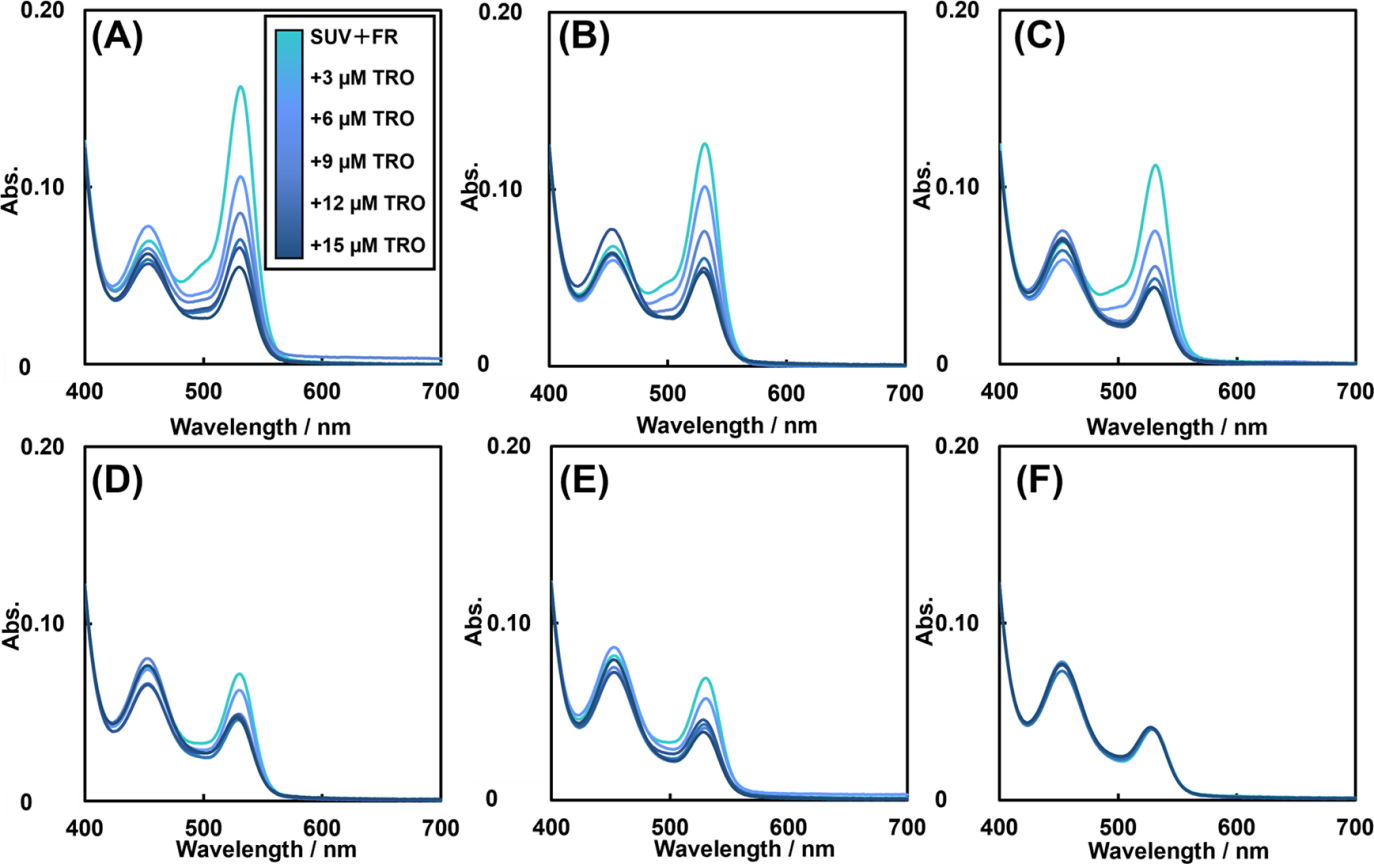
TBARS spectra of SUV suspensions subjected to lipid peroxidation in the presence of each concentration of LID and TRO. (A) 0 mM LID, (B) 0.64 mM LID, (C) 1.28 mM LID, (D) 1.96 mM LID, (E) 2.56 mM LID, and (F) 3.2 mM LID in [Fe(NH_4_)_2_(SO_4_)_2_] = 0.2 mM, [H_2_O_2_] = 0.1 mM, 10% ethanol in D.PBS, and 1.3 μg/mL phosphorus concentration SUV at 37 °C.

**Figure 3:**
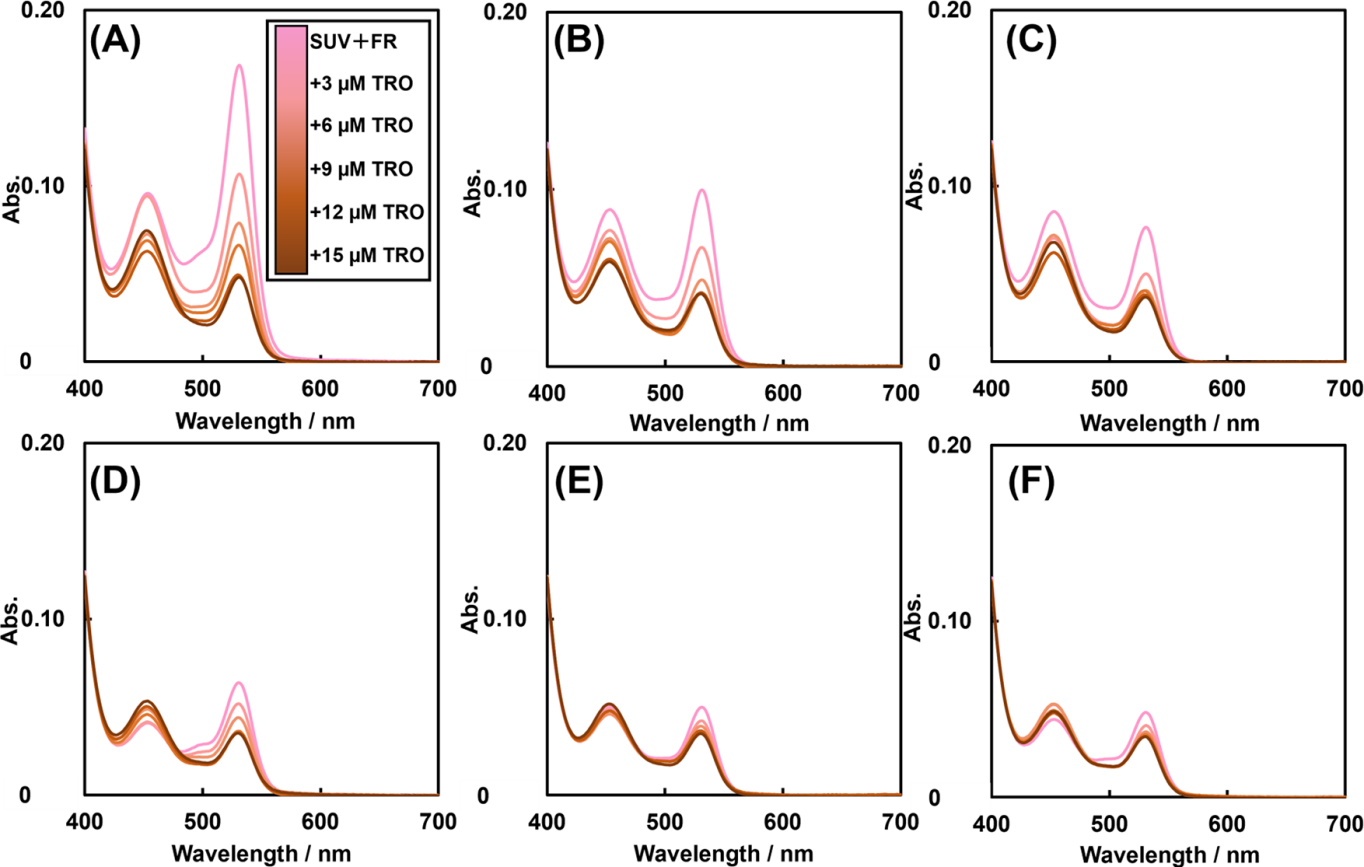
TBARS spectra of SUV suspensions subjected to lipid peroxidation in the presence of each concentration of DIB and TRO.(A) 0 mM DIB, (B) 0.4 mM DIB, (C) 0.8 mM DIB, (D) 1.2 mM DIB, (E) 1.6 mM DIB, and (F) 2.0 mM DIB in [Fe(NH_4_)_2_(SO_4_)_2_] = 0.2 mM, [H_2_O_2_] = 0.1 mM, 10% ethanol in D.PBS, and 1.3 μg/mL phosphorus concentration SUV at 37 °C.

To observe the effects of LID on the inhibition of lipid peroxidation by TRO, lipid peroxidation was induced by FR after adding various concentrations of TRO to a suspension of an SUV containing various concentrations of LID. Figure 2 shows the spectra of the supernatant obtained from the reaction of TBARS generated by lipid peroxidation with TBA. Figure 2 shows that both LID and TRO decreased the absorbance at 530 nm in a concentration-dependent manner. That is because TRO inhibits lipid peroxidation due to its radical-scavenging activity^30,32^ and LID improves the fluidity of liposomes and increases the distance between phospholipids, possibly making it more difficult to establish cascades.^26,33^ Absorbance at 455 nm appears to decrease in a LID concentration-dependent manner. However, some spectra show absorbance at 455 nm regardless of concentration. The possible reason can be that the absorbance at 455 nm was the sum of the absorbance peaks of SUV + FR, FR-only, TRO + FR, and the dye formed by the reaction of TBA with each of the products of SUV + FR. Thus, dyes that show absorbance at 455 nm are diverse and complicated. Therefore, a simple quantitative relationship between absorbance and drug concentration cannot be established. In conclusion, LID inhibits lipid peroxidation, and the absorbance at 455 nm was not concentration-dependent for either TRO or LID. The same experiment was performed with DIB as a control for LID, and Figure 3 shows the spectra of the supernatant obtained from the reaction of TBARS and TBA generated by lipid peroxidation in the SUV suspension with DIB. DIB, similar to LID, decreased the absorbance at 530 nm. Therefore, DIB inhibited lipid peroxidation. DIB had a higher log *P* value than LID and can be more easily incorporated into lipid membranes. Therefore, with DIB, inhibition of lipid peroxidation may have occurred at lower concentrations. These results indicate that LA inhibited lipid peroxidation specifically at lower concentration of DIB than that of LID.

### SVD of UV–Vis Spectra

Based on these results, we quantitatively analyzed the effect of LA on the inhibition of lipid peroxidation in TRO. Specifically, we used an analysis technique called SVD to extract the elements necessary to explain the data. SVD is a method to decompose all measured spectral data into several matrices and extract common characteristic components. The first component has absorbances at 530 and 455 nm in the same direction. Therefore, the first component is considered to reflect the average of the singular value-resolved spectral data. The second component has a peak absorbance at 530 nm and a negative peak at 455 nm. The third component has an absorbance peak at 455 nm and may not be derived from lipid peroxidation.

### Quantitative Evaluation of the Effect of LA Determined by the Inhibitory Activity by TRO to Lipid Peroxidation

**Figure 4:**
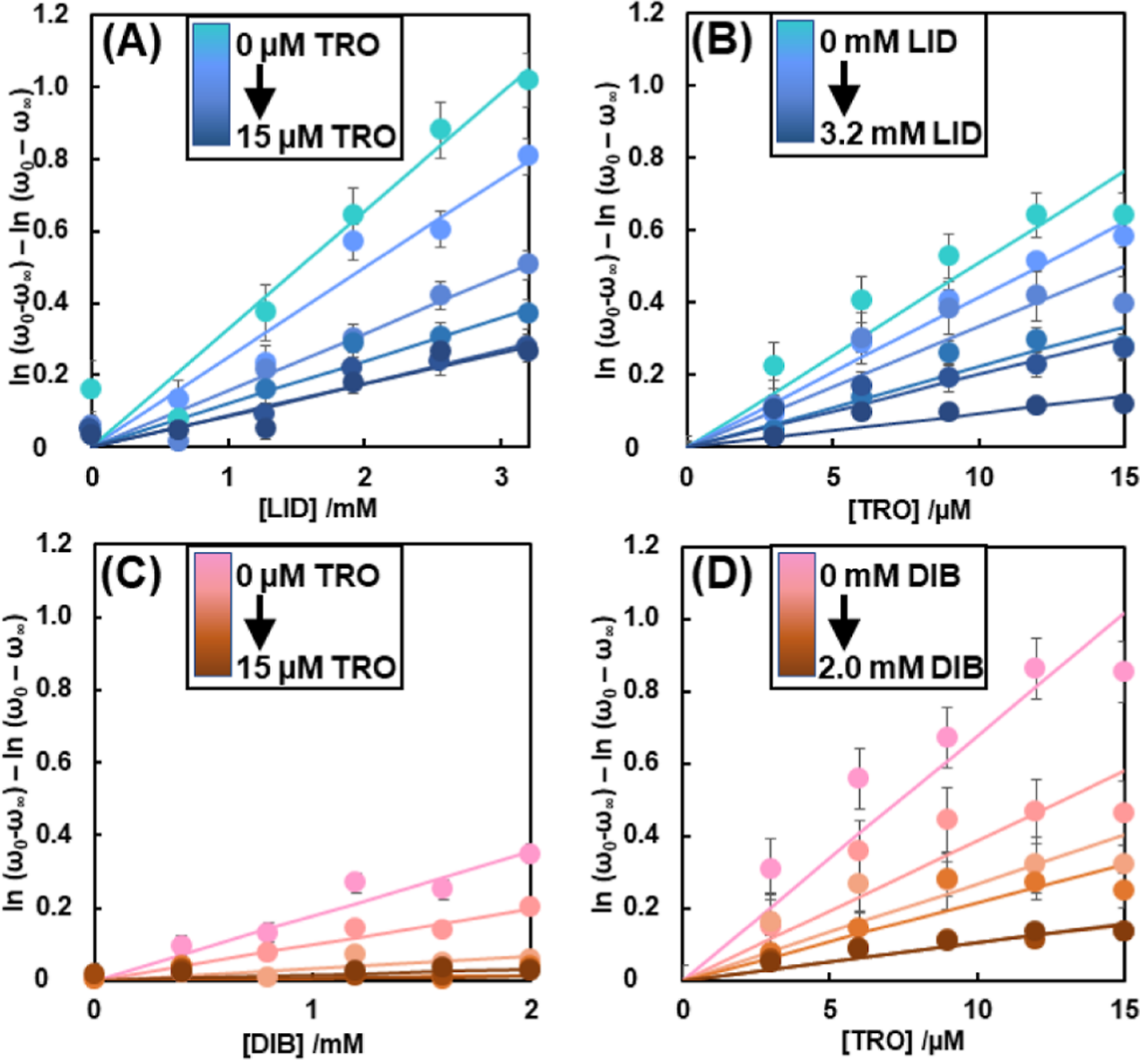
Plot of synthetic variate ω for each drug concentration. ω is the sum of the first and second principal components calculated by SVD. s and v are obtained as a result of SVD processing for the vis spectrum shown in Figures 1, 2, and S3–S5. (A) Relationship between LID and ω under each concentration of TRO. (B) Relationship between TRO and ω under each concentration of LID. (C) Relationship between DIB and ω under each concentration of TRO. (D) Relationship between TRO and ω under each concentration of DIB.

**Figure 5:**
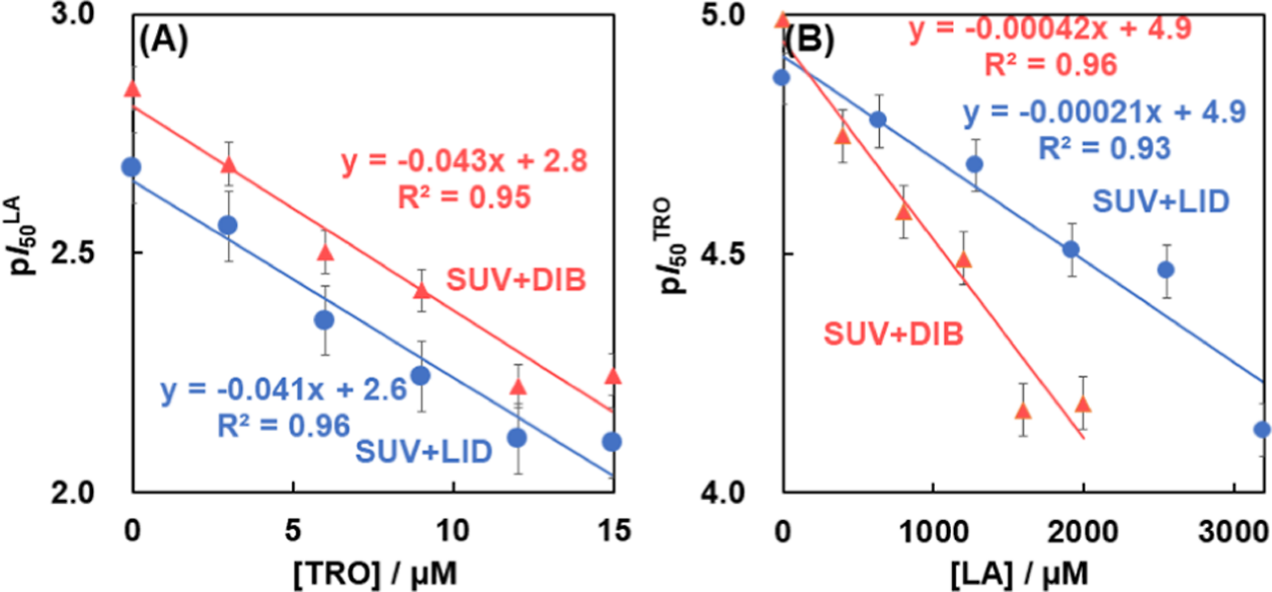
Diagrams of the p*I*_50_ of each drug calculated from *K* obtained from Figure 4 and eq 3 corresponding to another drug concentration. (A) Effect of LA on p*I*_50_^TRO^. There was no significant difference in p*I*_50_^LA^/[TRO]. (B) Effect of TRO on p*I*_50_^LA^. p*I*_50_^drug^ was calculated from eq 3.

Based on the above analysis, only the components derived from lipid peroxidation were extracted and evaluated using the sum of the first and second components, ω. Figure 4 shows the absorbance of the first component on the horizontal axis and the absorbance of the second component on the vertical axis. Figure 4 plots the drug concentrations on the horizontal axis and values calculated from curve fitting using eq 5 on the vertical axis. The effects of LA on the inhibitory activity of TRO were evaluated by calculating p*I*_50_. Figure 5A shows a graph evaluating p*I*_50_^LA^ against TRO. The slopes indicate that TRO reduced p*I*_50_^LA^ by the same amount for each p*I*_50_^LA^. The reductions were not significantly different. The reason behind TRO reducing the p*I*_50_^LA^, despite influencing a different cascade of reactions compared to LA, could be that LA affects the radical-scavenging activity of TRO. Therefore, to examine the possibility that LA affects the radical-scavenging activity of TRO, ESR measurements using DPPH radicals under various drug conditions were performed. The DPPH radical is stable at room temperature and has a unique ESR spectrum. Figure S6B–D shows graphs showing the TRO concentration-dependent decrease in DPPH radicals under each LA condition. In order to quantitatively evaluate this result, Figure S7 shows the results of SVD according to the previous method. From this result, we focused only on the first component and tracked the spectral change of the DPPH radical. Figure S8D shows the results in the presence of each LA. This is a graph showing the degree of DPPH reduction by TRO. There was no significant difference in the value of *d*[DPPH]/*d*[TRO], which indicates the degree of DPPH reduction due to TRO, under each LA condition. This result indicates that LA does not affect the radical-scavenging activity of TRO. One of the possible reasons can be the way the radical-scavenging activity of TRO is affected. Previous studies have shown that TRO scavenges radicals through the single-electron-transfer reaction.^25^ Therefore, TRO reacts with hydroxyl radicals required for the initiation reaction prior to lipid peroxidation. We examined the effect of TRO on the fluidity of cell membranes. However, it is considered that TRO does not affect the fluidity of cell membranes. There are three reasons why TRO was used as a radical-scavenging agent. First, TRO is a standard substance with radical-scavenging activity and is widely used in general. The second reason is that it is a derivative of α-Toc. Since α-Toc has a membrane-protective function in vivo, TRO, an α-Toc derivative, was used in this study. The third reason is that α-Toc has been reported to reduce the fluidity of cell membranes above the phase transition temperature.^34−36^ α-Toc behaves like cholesterol. Therefore, it is possible that the effect of improved LA fluidity on membrane protection cannot be observed accurately. For these reasons, it is considered that TRO does not affect the fluidity of the cell membrane. Figure 5B shows the p*I*_50_^TRO^ values for LA. Both LID and DIB decreased p*I*_50_^TRO^ in a concentration-dependent manner. In addition, DIB decreased p*I*_50_^TRO^ 1.9 times more than LID. We consider the reason why LA reduced p*I*_50_^TRO^. LA may improve the fluidity of the membrane,^30^ which may facilitate the migration of TRO from the membrane to the liquid phase. As a result, TRO is less likely to suppress lipid peroxidation within the lipid membrane, possibly resulting in a decrease in p*I*_50_^TRO^. Next, we focused on the log *P* values of each LA as the cause of this difference between the effects of DIB and LID: log *P* of DIB is 4.20 and that of LID is 2.20. Therefore, DIB is more hydrophobic than LID and is more easily incorporated into lipid membranes. Thus, DIB was found to be more effective than LID in this study. Therefore, we succeeded in separating the independent characteristics of LID and DIB in this experimental setting. The results showed that LA reduced the inhibition of lipid peroxidation by TRO, and LAs caused a hydrophobicity-dependent attenuation of inhibition of lipid peroxidation.

## Conclusions

First, LA was found to inhibit lipid peroxidation. DIB inhibited lipid peroxidation at a lower concentration than LID. Second, we used SVD to remove dyes with a peak absorbance at 455 nm, which is co-occurring with absorbance at 530 nm. As a result, we succeeded in extracting only the amount of dye derived from lipid peroxidation. The results indicated that LA reduced the inhibition of lipid peroxidation by TRO, and LAs caused a hydrophobicity-dependent attenuation of inhibition of lipid peroxidation. From this study, we were able to clarify the molecular mechanism by observing the dynamic structure of the liposome membrane and the drug as molecular arrangement and intermolecular interaction based on the above analysis. Furthermore, the studies that evaluate the direct action of drugs and biological membranes can provide a rational approach for elucidating the molecular mechanisms and pharmacological actions of various drugs and for efficient drug discovery and development.

## Supporting Information Available

TBARS spectra of MLV suspensions subjected to lipid peroxidation in the presence of each concentration of LID and TRO, TBARS spectra of MLV suspensions subjected to lipid peroxidation in the presence of each concentration of DIB and TRO, singular values (*s*_i_) and basis vectors (ψ_i_) obtained as a result of SVD processing for the vis spectra, plot of synthetic variate ω for each drug concentration, diagrams of p*I*_50_ of each drug, representative ESR spectra of the DPPH radical, singular values (*s*_i_) and basis vectors (ψ_i_) obtained as a result of SVD processing for the ESR spectra shown in Figure S6, and calculation of DPPH amount by SVD and the effect of LID and DIB on the radical-scavenging activity of TRO (PDF)

## Abbreviations

TRO: Trolox
LA: local anesthetic
LID: lidocaine
DIB: dibucaine
ROS: reactive oxygen species
PUFA: polyunsaturated fatty acid
α-Tocopherol: α-Toc
EyPC: egg yolk lecithin
DPPH: 1,1-diphenyl-2-picrylhydrazyl free radical
TBA: 2-thiobarbituric acid
BHT: 2,6-di-*tert*-butyl-*p*-cresol
MLV: multilamellar liposome
SUV: small unilamellar liposome
SVD: singular value decomposition
FR: Fenton reagent
PUFA: polyunsaturated fatty acid
MDA: malondialdehyde

## References

[ref1] Ferreira C. A.; Ni D.; Rosenkrans Z. T.; Cai W. Scavenging of reactive oxygen and nitrogen species with nanomaterials. Nano Res. 2018, 11, 4955–4984. 10.1007/s12274-018-2092-y. 30450165 PMC6233906

[ref2] Lundgren C. A. K.; Sjöstrand D.; Biner O.; Bennett M.; Rudling A.; Johansson A. L.; Brzezinski P.; Carlsson J.; von Ballmoos C.; Högbom M. Scavenging of superoxide by a membrane-bound superoxide oxidase. Nat. Chem. Biol. 2018, 14, 788–793. 10.1038/s41589-018-0072-x. 29915379 PMC6379055

[ref3] Su L. J.; Zhang J. H.; Gomez H.; Murugan R.; Hong X.; Xu D.; Jiang F.; Peng Z. Y. Reactive oxygen species-induced lipid peroxidation in apoptosis, autophagy, and ferroptosis. Oxid. Med. Cell Longev. 2019, 2019, 1–13. 10.1155/2019/5080843. PMC6815535 31737171

[ref4] NIKI E. Antioxidants in relation to lipid peroxidation. Chem. Phys. Lipids 1987, 44, 227–253. 10.1016/0009-3084(87)90052-1. 3311418

[ref5] Ernster L.; Nordenbrand K. Microsomal lipid peroxidation. Meth. Enzymol. 1967, 10, 574–580. 10.1016/0076-6879(67)10099-2.

[ref6] Halliwell B. Lipid peroxidation, antioxidants and cardiovascular disease: How should we move forward?. Cardiovasc. Res. 2000, 47, 410–418. 10.1016/s0008-6363(00)00097-3. 10963714

[ref7] Yuen K. S.; Halliday G. M. α-Tocopherol, an Inhibitor of Epidermal Lipid Peroxidation, Prevents Ultraviolet Radiation from Suppressing the Skin Immune System. Photochem. Photobiol. 1997, 65, 587–592. 10.1111/j.1751-1097.1997.tb08610.x. 9077145

[ref8] Yin H.; Xu L.; Porter N. A. Free radical lipid peroxidation: Mechanisms and analysis. Chem. Rev. 2011, 111, 5944–5972. 10.1021/cr200084z. 21861450

[ref9] Gautam R.; Jachak S. M. Recent developments in anti-inflammatory natural products. Med. Res. Rev. 2009, 29, 767–820. 10.1002/med.20156. 19378317

[ref10] Jovanović A. A.; Balanč B. D.; Ota A.; Ahlin Grabnar P.; Djordjević V. B.; Šavikin K. P.; Bugarski B. M.; Nedović V. A.; Poklar Ulrih N. Comparative Effects of Cholesterol and β-Sitosterol on the Liposome Membrane Characteristics. Eur. J. Lipid Sci. Technol. 2018, 120, 1800039 10.1002/ejlt.201800039.

[ref11] Boiteux C.; Vorobyov I.; French R. J.; French C.; Yarov-Yarovoy V.; Allen T. W. Local anesthetic and antiepileptic drug access and binding to a bacterial voltage-gated sodium channel. Proc. Natl. Acad. Sci. U.S.A. 2014, 111, 13057–13062. 10.1073/pnas.1408710111. 25136136 PMC4246943

[ref12] Weizenmann N.; Huster D.; Scheidt H. A. Interaction of local anesthetics with lipid bilayers investigated by 1H MAS NMR spectroscopy. Biochim. Biophys. Acta, Biomembr. 2012, 1818, 3010–3018. 10.1016/j.bbamem.2012.07.014. 22842001

[ref13] Paiva J. G.; Paradiso P.; Serro A. P.; Fernandes A.; Saramago B. Interaction of local and general anaesthetics with liposomal membrane models: A QCM-D and DSC study. Colloids Surf., B 2012, 95, 65–74. 10.1016/j.colsurfb.2012.02.027. 22424911

[ref14] Tsuchiya H.; Mizogami M. Interaction of local anesthetics with biomembranes consisting of phospholipids and cholesterol: Mechanistic and clinical implications for anesthetic and cardiotoxic effects. Anesthesiol. Res. Pract. 2013, 2013, 1–18. 10.1155/2013/297141. PMC3794646 24174934

[ref15] Kanai Y.; Katsuki H.; Takasaki M. Lidocaine disrupts neuronal membrane of rat sciatic nerve *in vitro*. Anesth. Analg. 2000, 91, 944–948. 10.1097/00000539-200010000-00033. 11004054

[ref16] Rutkowska M.; Trocha M.; Szandruk M.; Słupski W.; Rymaszewska J. Effects of supplementation with fish oil and n-3 pufas enriched egg yolk phospholipids on anhedonic-like response and body weight in the rat chronic mild stress model of depression. Pharmazie 2013, 68, 685–688. 24020125

[ref17] Kunimoto M.; Inoue K.; Nojima S. Effect of ferrous ion and ascorbate-induced lipid peroxidation on liposomal membranes. Biochim. Biophys. Acta Biomembr. 1981, 646, 169–178. 10.1016/0005-2736(81)90284-4. 6168284

[ref18] Fukuzawa K.; Tokumura A.; Ouchi S.; Tsukatani H. Antioxidant activities of tocopherols on Fe^2+^-ascorbate-induced lipid peroxidation in lecithin liposomes. Lipids 1982, 17, 511–513. 10.1007/bf02535334. 7121213

[ref19] Ohyashiki T.; Karino T.; Matsui K. Stimulation of Fe^2+^-induced lipid peroxidation in phosphatidylcholine liposomes by aluminum ions at physiological pH. Biochim. Biophys. Acta 1993, 1170, 182–188. 10.1016/0005-2760(93)90069-L. 8399343

[ref20] Mondal S.; Basu S.; Mandal D. Ground- and Excited-State Proton-Transfer Reaction of 3-Hydroxyflavone in Aqueous Micelles. Chem. Phys. Lett. 2009, 479, 218–223. 10.1016/j.cplett.2009.08.026.

[ref21] Bacellar I. O. L.; Baptista M. S. Mechanisms of Photosensitized Lipid Oxidation and Membrane Permeabilization. ACS Omega 2019, 4, 21636–21646. 10.1021/acsomega.9b03244. 31891041 PMC6933592

[ref22] Yoshida K.; Terao J.; Suzuki T.; Takama K. Inhibitory effect of phosphatidylserine on iron-dependent lipid peroxidation. Biochem. Biophys. Res. Commun. 1991, 179, 1077–1081. 10.1016/0006-291x(91)91929-7. 1898388

[ref23] Ames B. Assay of inorganic phosphate, total phosphate and phosphatase. Methods Enzymol. 1966, 8, 115–118. 10.1016/0076-6879(66)08014-5.

[ref24] Minotti G.; Aust S. D. The requirement for iron (III) in the initiation of lipid peroxidation by iron (II) and hydrogen peroxide. J. Biol. Chem. 1987, 262, 1098–1104. 10.1016/s0021-9258(19)75755-x. 3027077

[ref25] Takatsuka M.; Goto S.; Kobayashi K.; Otsuka Y.; Shimada Y. Evaluation of pure antioxidative capacity of antioxidants: ESR spectroscopy of stable radicals by DPPH and ABTS assays with singular value decomposition. Food Biosci. 2022, 48, 101714 10.1016/j.fbio.2022.101714.

[ref26] Bakonyi M.; Berkó S.; Budai-Szűcs M.; Kovács A.; Csányi E. DSC for evaluating the encapsulation efficiency of lidocaine-loaded liposomes compared to the ultracentrifugation method. J. Therm. Anal. Calorim. 2017, 130, 1619–1625. 10.1007/s10973-017-6394-1.

[ref27] Shiratori T.; Goto S.; Sakaguchi T.; Kasai T.; Otsuka Y.; Higashi K.; Makino K.; Takahashi H.; Komatsu K.; Komatsu K. Singular value decomposition analysis of the secondary structure features contributing to the circular dichroism spectra of model proteins. Biochem. Biophys. Rep. 2021, 28, 101153 10.1016/j.bbrep.2021.101153. 34712848 PMC8528683

[ref28] Steinbock O.; Neumann B.; Cage B.; Saltiel J.; Müller S. C.; Dalal N. S. A demonstration of principal component analysis for epr spectroscopy: Identifying pure component spectra from complex spectra. Anal. Chem. 1997, 69, 3708–3713. 10.1021/ac970308h.

[ref29] Janero D. R. Malondialdehyde and thiobarbituric acid-reactivity as diagnostic indices of lipid peroxidation and peroxidative tissue injury. Free Radic. Biol. Med. 1990, 9, 515–540. 10.1016/0891-5849(90)90131-2. 2079232

[ref30] Bektaşoğlu B.; Esin Çelik S.; Özyürek M.; Güçlü K.; Apak R. Novel hydroxyl radical scavenging antioxidant activity assay for water-soluble antioxidants using a modified CUPRAC method. Biochem. Biophys. Res. Commun. 2006, 345, 1194–1200. 10.1016/j.bbrc.2006.05.038. 16716257

[ref31] Hiroshige R.; Goto S.; Tsunoda C.; Ichii R.; Shimizu S.; Otsuka Y.; Makino K.; Takahashi H.; Yokoyama H. Trajectory of the spectral/structural rearrangements for photo-oxidative reaction of neat ketoprofen and its cyclodextrin complex. J. Incl. Phenom. Macrocycl. Chem. 2022, 102, 791–800. 10.1007/s10847-022-01160-3.

[ref32] Lúcio M.; Nunes C.; Gaspar D.; Ferreira H.; Lima J. L. F. C.; Reis S. Antioxidant Activity of Vitamin E and Trolox: Understanding of the Factors That Govern Lipid Peroxidation Studies In Vitro. Food Biosci. 2009, 4, 312–320. 10.1007/s11483-009-9129-4.

[ref33] Tsuchiya H.; Mizogami M. Comparative interactions of anesthetic alkylphenols with lipid membranes. Open J. Anesthesiol. 2014, 04, 308–317. 10.4236/ojanes.2014.412044.

[ref34] Tsuchiya K.; Nakanishi H.; Sakai H.; Abe M. Temperature-dependent vesicle formation of aqueous solutions of mixed cationic and anionic surfactants. Langmuir 2004, 20, 2117–2122. 10.1021/la0302908. 15835660

[ref35] Arora A.; Byrem T. M.; Nair M. G.; Strasburg G. M. Modulation of Liposomal Membrane Fluidity by Flavonoids and Isoflavonoids. Arch. Biochem. Biophys. 2000, 373, 102–109. 10.1006/abbi.1999.1525. 10620328

[ref36] Berlin E.; Bhathena S. J.; Judd J. T.; Nair P. P.; Peters R. C.; Bhagavan H. N.; Belcher B. R.; Taylor P. R. Effects of omega-3 fatty acid and vitamin E supplementation on erythrocyte membrane fluidity, tocopherols, insulin binding, and lipid composition in adult men. J. Nutr. Biochem. 1992, 3, 392–400. 10.1016/0955-2863(92)90013-9.

