# Supplementary material for: Effects of Local Anesthetics on Liposomal Membranes Determined by Their Inhibitory Activity of Lipid Peroxidation
Source: Mol Pharm. 2023 Apr 27;20(6):2911–8. doi: 10.1021/acs.molpharmaceut.2c01053 (PMC10245376; doi:10.1021/acs.molpharmaceut.2c01053)
Supplement: Supplementary file 1 — mp2c01053_si_001.pdf [file mp2c01053_si_001.pdf]

SUPPORTING INFORMATION

Effects of local anesthetics on liposomal membranes deter-mined by their inhibitory activity of lipid peroxidation

Yusuke Horizumi, Satoru Goto, Miwa Takatsuka, Hideshi Yokoyama

Faculty of Pharmaceutical Sciences, Tokyo University of Science,
2641 Yamazaki, Noda, Chiba, 278-8510, Japan.

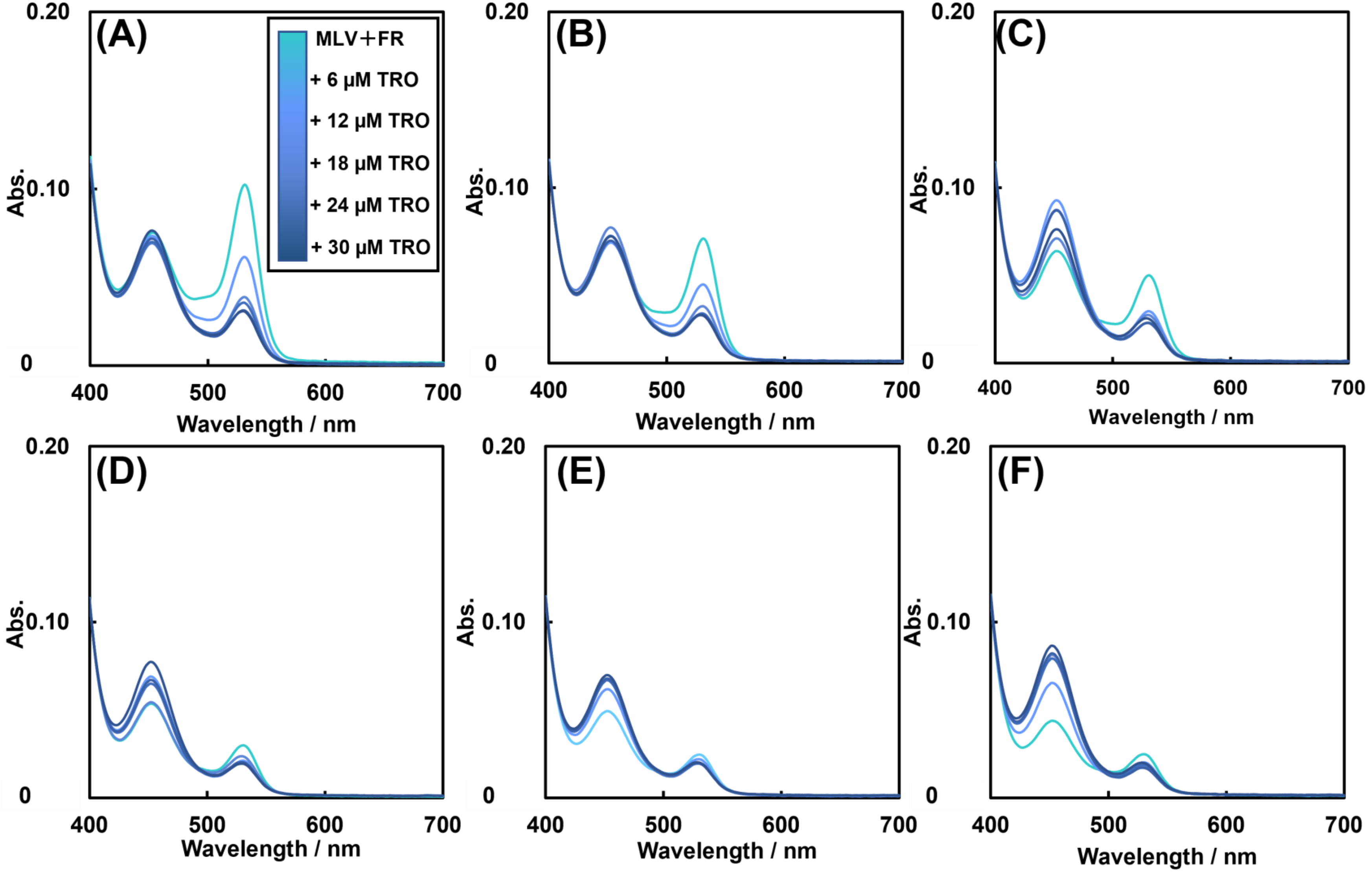

Fig. S1. TBARS spectra of MLV suspensions subjected to lipid peroxidation in the presence of each concentration of LID and TRO. (A) 0 mM LID, (B) 1.28 mM LID, (C) 2.56 mM LID, (D) 3.94 mM LID, (E) 5.12 mM LID, (F) 6.4 mM LID, in $[Fe(NH_4)_2(SO_4)_2]$ = 0.2 mM, $[H_2O_2]$ = 0.1 mM, 10% ethanol in D.PBS, 1.3 μg / mL Phosphorus concentration MLV at 37°C.

To investigate whether LID has a similar effect on TRO-induced inhibition of lipid peroxidation in MLV suspension, we induced lipid peroxidation by FR after adding various concentrations of TRO to a suspension of SUV containing different concentrations of LID. The same method and measurement procedure for lipid peroxidation were used as described in the text. As shown in Fig. S1, both TRO and LID exhibited concentration-dependent decreases in absorbance at 530 nm, even under MLV suspension conditions. Absorbance at 455 nm also appeared to decrease in a LID concentration-dependent manner, although some spectra showed absorbance at 455 nm irrespective of concentration. These results suggest that the effects of TRO and LID under MLV suspension conditions are similar to those observed under SUV suspension conditions. However, the effects of LID and TRO were weaker under the MLV suspension condition than under the SUV suspension condition.

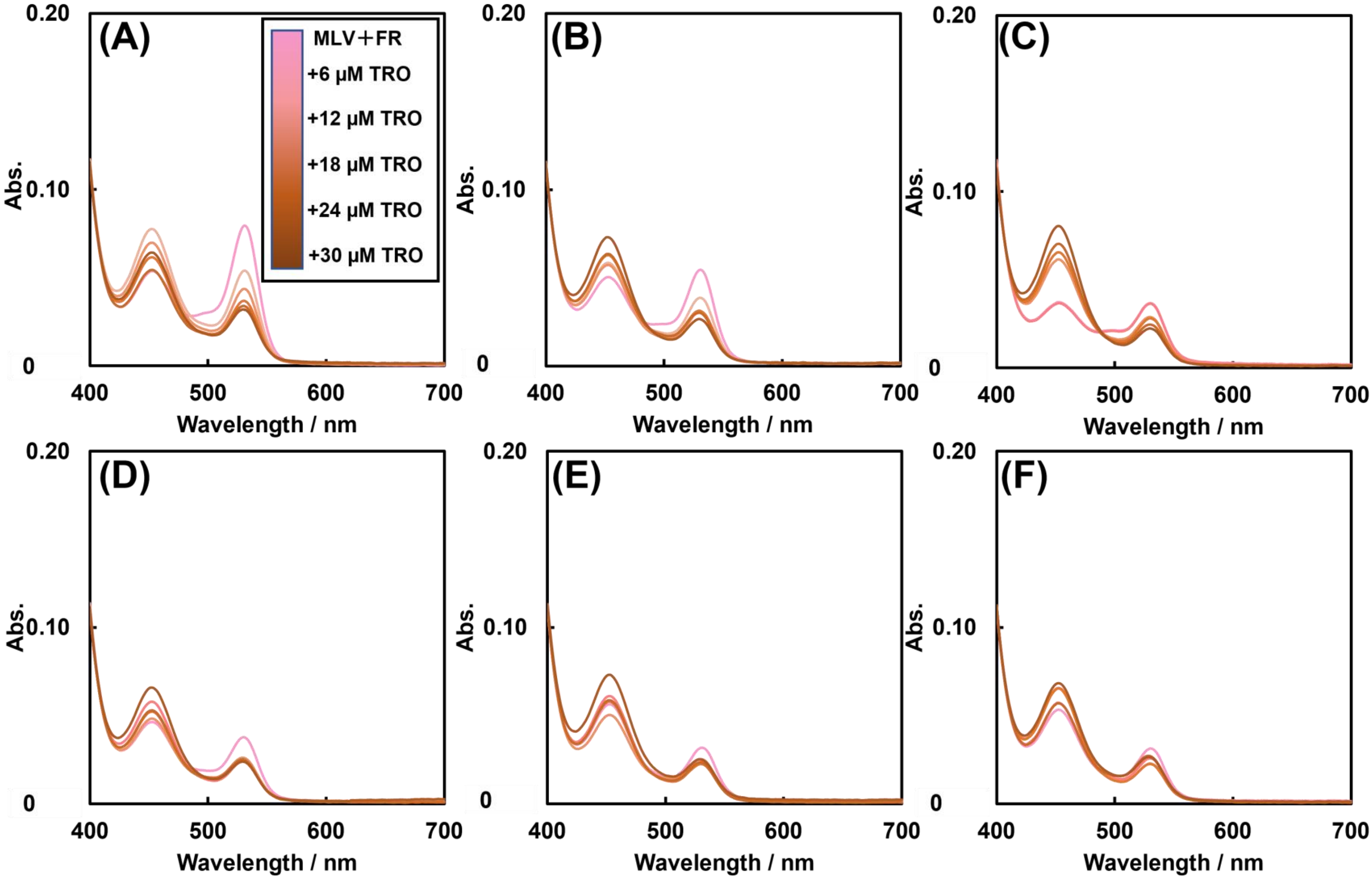

Fig. S2. TBARS spectra of MLV suspensions subjected to lipid peroxidation in the presence of each concentration of DIB and TRO.(A) 0 mM DIB, (B) 0.8 mM DIB, (C) 1.6 mM DIB, (D) 2.4 mM DIB, (E) 3.2 mM DIB, (F) 4.0 mM DIB, in [$Fe(NH_4)_2(SO_4)_2$] = 0.2 mM, [H_2O_2] = 0.1 mM, 10% ethanol in D.PBS, 1.3 μg / mL Phosphorus concentration MLV at 37℃.

To investigate whether the inhibitory effect of TRO-induced lipid peroxidation is affected by DIB in MLV suspensions, FR was used to induce lipid peroxidation after adding various concentrations of TRO to an SUV suspension containing different concentrations of DIB. The method and measurement techniques for lipid peroxidation were identical to those described in the text. Figure S2 shows that both TRO and DIB caused a concentration-dependent decrease in absorbance at 530 nm, even under MLV suspension conditions. Absorbance at 455 nm appeared to decrease with increasing DIB concentration, although some spectra still exhibited absorbance at 455 nm regardless of concentration. These findings indicate that the effects of TRO and DIB under MLV suspension conditions are similar to those observed under SUV suspension conditions. However, the effects of DIB and TRO were weaker under the MLV suspension condition than under the SUV suspension condition.

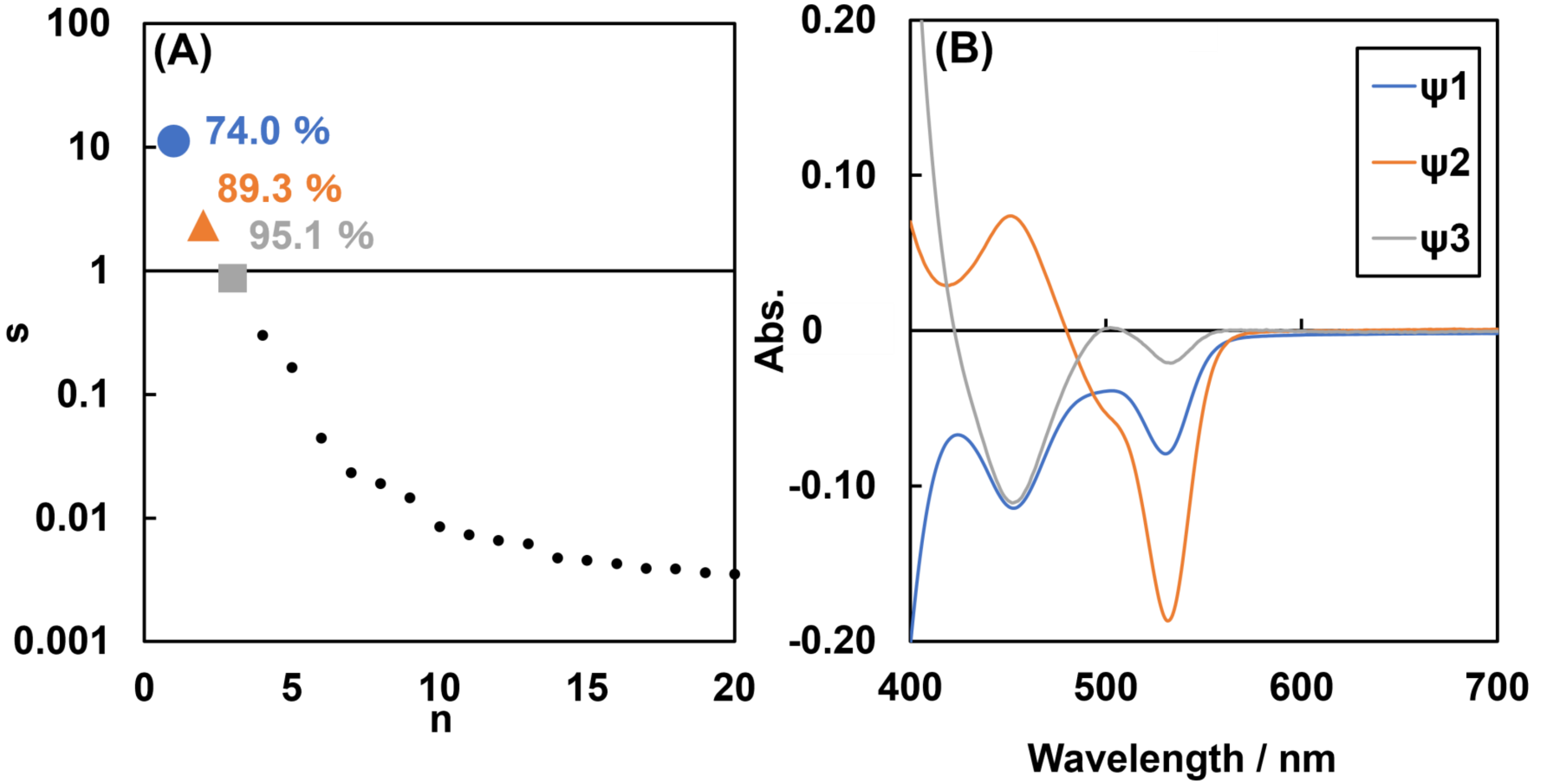

Fig. S3. (A): Singular values (s_i) and (B): basis vectors (ψ_i) obtained as a result of SVD processing for the Vis spectra shown in Fig. 1, Fig. 2, Fig. 3, Fig. S1, and Fig. S2.

To quantitatively analyze the effect of LA on the inhibition of TRO-induced lipid peroxidation, we utilized Singular Value Decomposition (SVD) to extract the necessary elements to explain the data. SVD is a method that decomposes all measured spectral data into several matrices and extracts common characteristic components. The same method as described in the text was performed, and Fig. S3(A) shows the magnitude of each component's singular value. We observed that up to the third component occupied 95% of all singular values. Fig. S3(B) displays the basis spectrum up to the third component. The first component exhibited absorbance at 530 nm and 455 nm in the same direction, reflecting the average of the singular value-resolved spectral data. The second component had a peak absorbance at 530 nm and a negative peak at 455 nm.

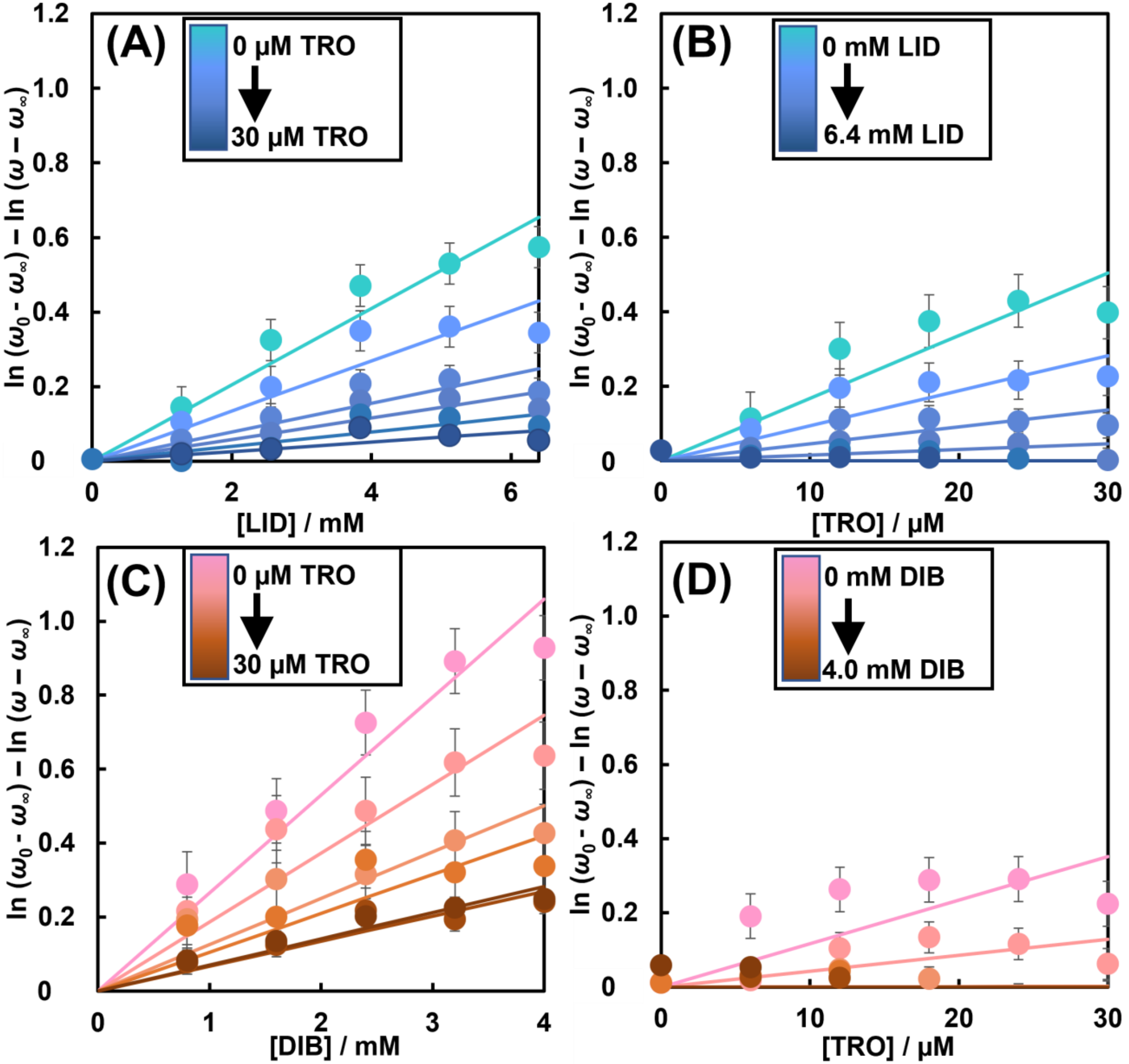

Fig. S4. The plot of synthetic variate ω for each drug concentration. ω is the sum of the first and second principal components calculated by SVD processing for the UV-Vis spectra shown in Fig. S1 and Fig. S2. (A) Relationship between LID and ω under each concentration of TRO (B) Relationship between TRO and ω under each concentration of LID (C) Relationship between DIB and ω under each concentration of TRO (D) Relationship between TRO and ω under each concentration of DIB

.

Based on Fig. S3, we extracted and evaluated only the components derived from lipid peroxidation using the sum of the first and second components, ω. Fig. S4 displays the absorbance of the first component on the horizontal axis and the absorbance of the second component on the vertical axis in MLV suspension. The effects of LA on the inhibitory activity of TRO was evaluated by calculating pI_{50}.

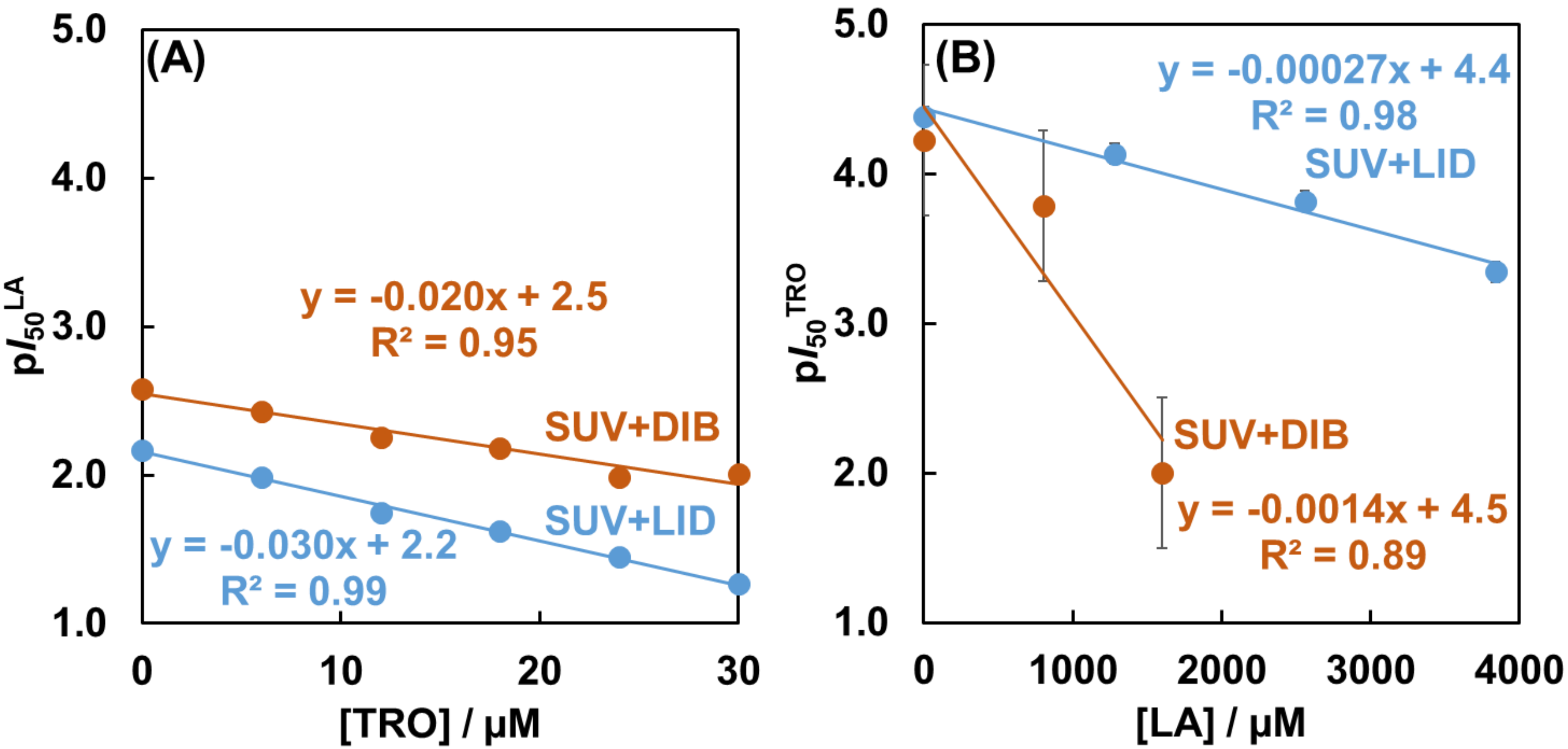

Fig. S5. The diagrams of the pI_{50} of each drug are calculated from K obtained from Fig. S4 and Eqn. 3 corresponding to another drug concentration. (A) Effect of LA on pI_{50}^{TRO} (B) Effect of TRO on pI_{50}^{LA}. pI_{50}^{drug} was calculated from Eqn. (3).

Based on Fig. S4, the effects of TRO on pI_{50}^{LA} and LA on pI_{50}^{TRO} in MLV suspension were plotted, respectively. In MLV suspension, TRO decreased pI_{50}^{TRO} and LA decreased pI_{50}^{TRO}, similar to SUV suspension. In addition, DIB reduced pI_{50}^{TRO} 1.9 times more than LID.

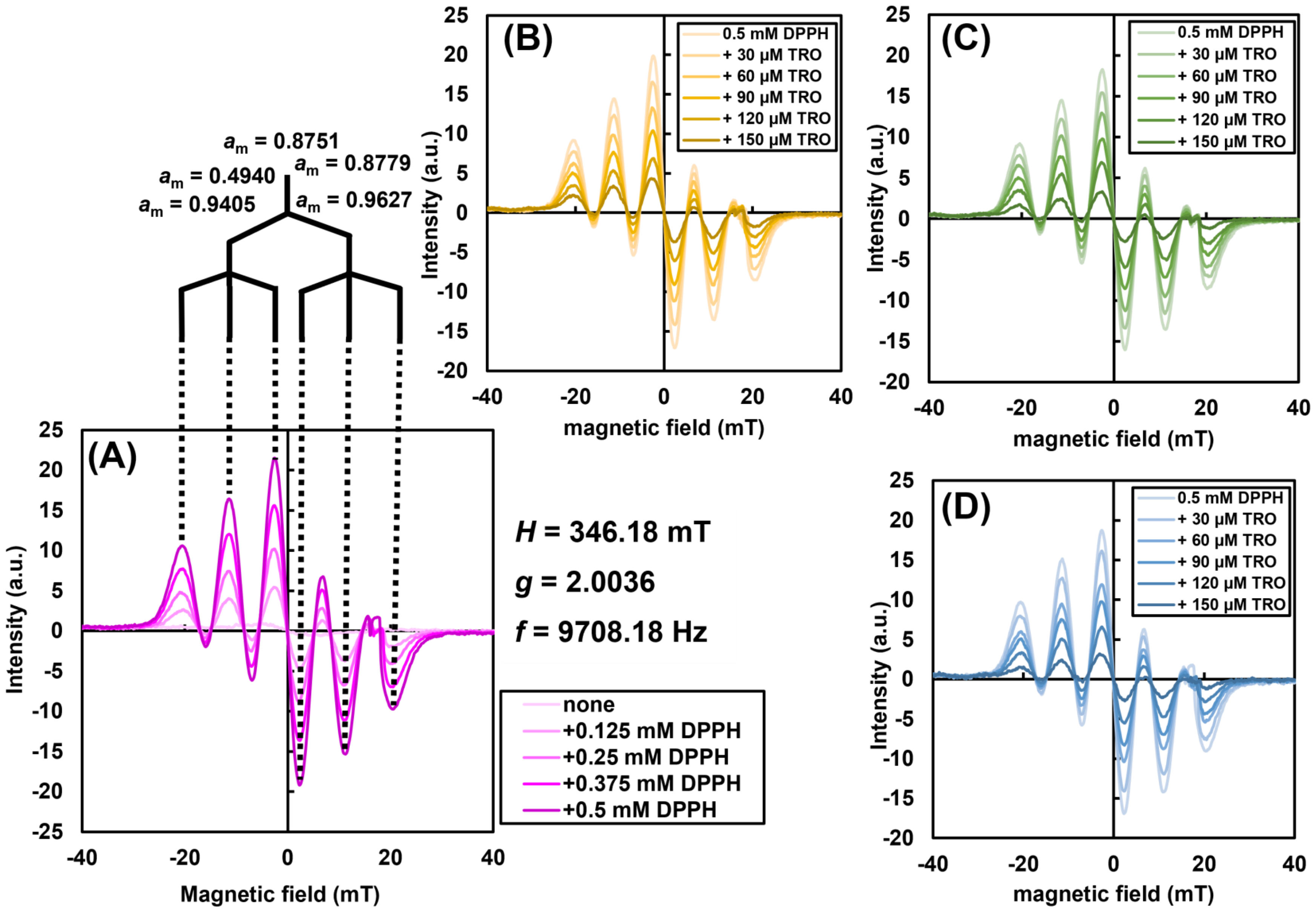

Fig. S6. Representative ESR Spectra of the DPPH Radical. (A) ESR spectra of DPPH radicals at each concentration (B), (C), and (D) were the ESR spectra obtained of the radical reaction generated by the DPPH assay using each drug. (B) none (C) 16 mM LID, (D) 10 mM DIB 20% (v/v) water in EtOH solution. [DPPH] = 0.5 mM, Trolox was added in each concentration.

To investigate the possibility that LA affects the radical scavenging activity of TRO, we performed ESR measurements using DPPH radicals under various drug conditions. The DPPH radical is stable at room temperature and exhibits a unique ESR spectrum. As shown in Fig S6(A), DPPH radicals increased the ESR spectrum specific to DPPH in a concentration-dependent manner. Fig. S6 (B), (C), and (D) are graphs showing the TRO concentration-dependent decrease in DPPH radicals under each LA condition.

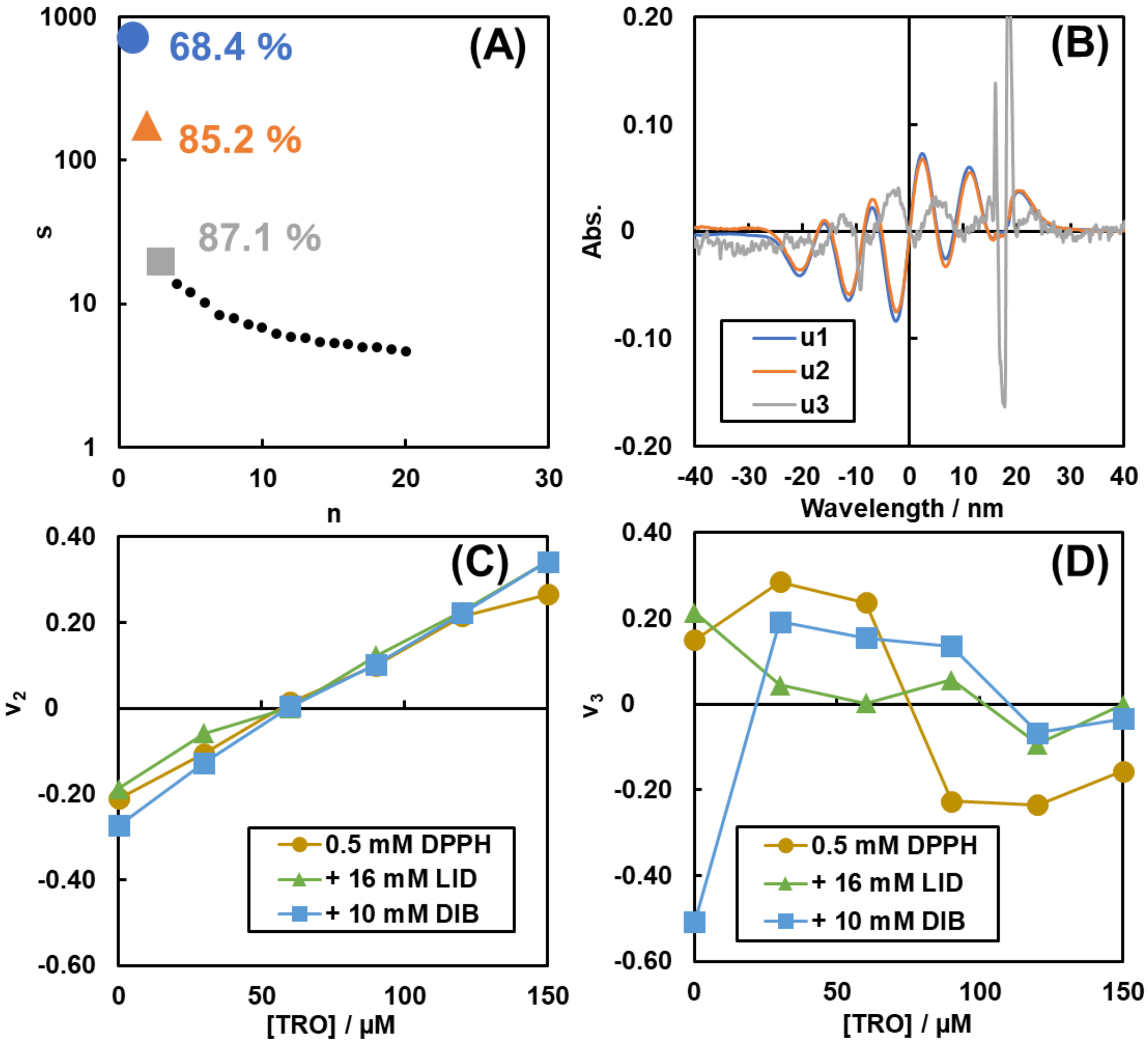

Fig. S7. (A): Singular values (s_i) and (B): basis vectors (ψ_i) obtained as a result of SVD processing for the ESR spectra shown in Fig. S6. (C) and (D) are interpretation of each component by SVD. variation of v_2 (C), and variation of v_3 (D) with respect to drug concentration.

To quantitatively evaluate Fig. S6, we used SVD according to the previous method, and Fig. S7 shows the results. Fig. S7(A) displays the magnitude of each component. Fig. S7(B) shows the basis spectrum of the third component. Fig. S7(C) shows that the singular vector of the second component decreased in a TRO concentration-dependent manner, similar to the singular vector of the first component. Fig. S7(D) shows that the singular vector of the third component changed independently of the TRO concentration.

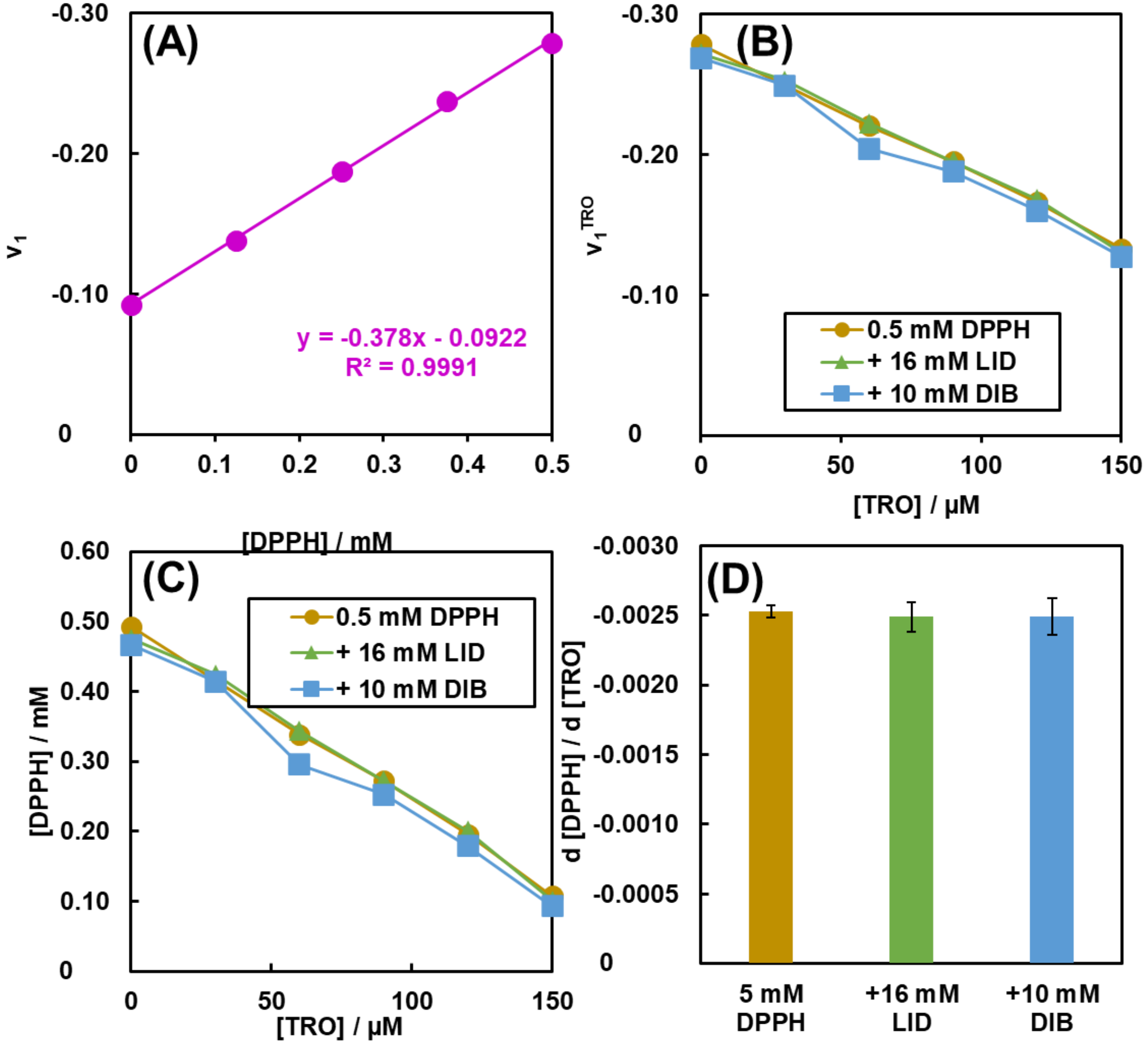

Fig. S8. (A) The plot of v_1 against [DPPH]. v_1 is obtained as a result of SVD processing for the ESR spectra shown in Fig. 5. (B) Interpretation of each component by SVD Variation of v_1. (C) The plot of TRO concentration and DPPH is calculated from Eqn. 4 when each LA is added. (D) There was no significant difference in d [DPPH] / d [TRO] under each condition.

Figure S8 depicts the results of the ESR spectra processing for v1 plotted against [DPPH]. The interpretation of each component using SVD and the variation of v_1 are presented in Fig. S8(B). Fig. S8(C) shows the TRO concentration and DPPH calculated from Eqn. 4 when each LA is added, and Fig. S8(D) shows the degree of DPPH reduction by TRO. The value of d [DPPH] / d [TRO], which reflects the degree of DPPH reduction due to TRO, was not significantly different under each LA condition.
